# MCL restrained ROS/AKT/ASAH1 pathway to therapy tamoxifen resistance breast cancer by stabilizing NRF2

**DOI:** 10.1111/cpr.13700

**Published:** 2024-06-26

**Authors:** Xiao Han, Yupeng Zhang, Yin Li, Zhoujun Lin, Zhenkun Fu, Changjun Wang, Shengjie Zhang, Di Shao, Chenggang Li

**Affiliations:** ^1^ Zhejiang Cancer Hospital Hangzhou Institute of Medicine (HIM), Chinese Academy of Sciences Hangzhou Zhejiang China; ^2^ Key Laboratory of Prevention Diagnosis and Therapy of Upper Gastrointestinal Cancer of Zhejiang Province Hangzhou China; ^3^ State Key Laboratory of Medicinal Chemical Biology and College of Pharmacy Nankai University Tianjin China; ^4^ Department of Immunology & Wu Lien‐Teh Institute & Heilongjiang Provincial Key Laboratory for Infection and Immunity Harbin Medical University & Heilongjiang Academy of Medical Science Harbin China; ^5^ Department of Breast Surgery Peking Union Medical College Hospital Beijing China; ^6^ Chonggang General Hospital Chongqing China; ^7^ Chongqing Emergency Medical Center Chongqing University Central Hospital Chongqing China

## Abstract

Tamoxifen resistance is a common and difficult problem in the clinical treatment of breast cancer (BC). As a novel antitumor agent, Micheliolide (MCL) has shown a better therapeutic effect on tumours; however, little is known about MCL and its role in BC therapy. With tamoxifen stimulation, drug‐resistant BC cells MCF7TAMR and T47DTAMR obtained a high oxidative status and Amidohydrolase 1 (ASAH1) was abnormally activated. The inhibition of ASAH1 rescued the sensitivity of resistant cells to tamoxifen. We found that MCL inhibited the expression of ASAH1 and cell proliferation, especially in MCF7TAMR and T47DTAMR cells. The high oxidative stress status of resistant cells stimulated the expression of ASAH1 by positively regulating AKT, which was restrained by MCL. MCL activated NRF2 by directly binding to KEAP1 and promoting the antioxidant level of tamoxifen‐resistant (TAMR) cells. In addition, ACT001, the prodrug of MCL, significantly inhibited the tumour growth of TAMR cells in preclinical xenograft tumour models. In conclusion, ASAH1 mediates tamoxifen resistance in ER‐positive BC cells. MCL could activate the cellular antioxidant system via NRF2/KEAP1 and inhibit ASAH1 expression through the ROS/AKT signalling pathway, thus suppressing cell proliferation. MCL could be used as a potential treatment for TAMR‐BC.

## Graphical abstract

ASAH1 mediates tamoxifen resistance in ER‐positive breast cancer cells. MCL could activate the cellular antioxidant system via NRF2/KEAP1 and inhibit ASAH1 expression by the ROS/AKT signalling pathway, thus suppressing cell proliferation in vitro and in vivo.

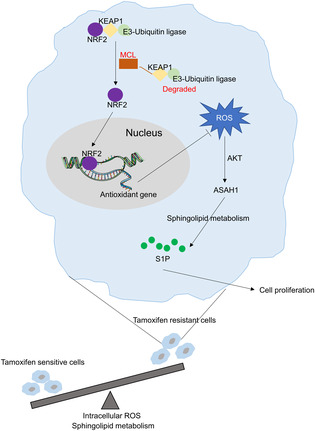

## 1 INTRODUCTION

Breast cancer (BC) is the second leading cause of death among all tumours in women, and ER‐positive BC accounts for approximately 70% of BC patients.^1^ Chemotherapy combined with endocrine therapy is an effective treatment for BC. However, drug resistance is a major obstacle in clinical treatment. With the progression of drug resistance, the patterns of surface receptors^2^ are changed, and the metabolic balance^1^ is disrupted in tumour cells. Especially for tamoxifen, a competitive inhibitor of estradiol,^3^ its long‐term treatment induced reactive oxygen species (ROS) accumulation in ER‐positive BC cells, which partially contributed to drug resistance. ROS plays a two‐sided role in cancer, where low levels of ROS may be beneficial, but excessive accumulation promotes tumour progression.^4^ While multiple pathways lead to ROS generation, the mitochondrial electron transport chain and NADPH oxidase (NOX) family enzymes are reported as major sources of ROS. Additionally, there are also some antioxidant proteins that maintain the balance of oxidative stress in tumour cells.^5^ Nuclear factor erythroid 2‐related factor (NRF2), a cytoprotective factor, is involved in the regulation of transcriptional expression, including antioxidant genes.^6^ Tamoxifen has been reported to induce ROS accumulation by reducing NRF2 expression, thus inducing impaired glycolysis and a survival advantage in gallbladder cancer.^7^

In addition to oxidative stress, the disruption of metabolic balance is also an important feature of drug‐resistant cells. We focused on the change of genes related to sphingolipid metabolism in this study. Amidohydrolase 1 (ASAH1) is a key enzyme of sphingolipid metabolism, which catalyses ceramide to sphingosine, and sphingosine further generates sphingosine 1 phosphate (S1P) under the catalysis of sphingosine kinase (SPHK). As a bioactive factor, S1P further binds to the S1P receptor (1–5), a family of G‐protein‐coupled receptors that couple with multiple G‐proteins to activate intracellular signalling pathways and promote tumour progression.^8^ A small molecule acid ceramidase inhibitor, ceranib 2, suppresses the proliferation of tumour cells, including ER‐positive BC cells.^9^ Carmofur, another small molecule inhibitor of ASAH1 in the clinic, significantly inhibits glioblastoma migration.^10^ However, carmofur still has not been approved by the FDA due to the higher incidence of leukoencephalopathy in the therapy process of hepatocellular carcinoma (HCC).^11^ In acute pancreatitis, ASAH1 accommodates the activity of trypsinogen through sphingosine, which binds to pyruvate kinase and reduces ROS production.^12^ On the other hand, ASAH1 depletion increases peroxisome‐derived ROS and inhibits melanoma growth.^13^ ASAH1 elevation has been found in a variety of cancers,^11^ but the specific molecular mechanism of ASAH1 upregulation and the relationship between ASAH1 and ROS remain unclear.

Given that there were no effective clinical inhibitors of ASAH/SPHK/S1PR signalling, we would like to explore possible drugs targeting sphingolipid metabolism or enhancing the sensitivity of drug‐resistant cells based on existing clinical drugs. DMAMCL was discovered through literature comparison and drug screening. Dimethylaminomicheliolide (DMAMCL, named MCL in brief) is an allosteric small molecule compound of parthenolide (PTL), and it is more stable and safer than PTL.^14^ As a novel antitumor agent, MCL does not injure normal cells but selectively targets cancerous cells.^15^ MCL therapy triggers ROS‐dependent apoptosis in HCC^16^ and rhabdomyosarcoma.^17^ However, whether MCL is effective in tamoxifen‐resistant (TAMR) tumour models remains to be further studied. In our work, we demonstrated the abnormal signature of ASAH1 in TAMR ER‐positive BC cells. MCL therapy activated the cellular antioxidant system via NRF2/KEAP1 and restrained ASAH1‐mediated tamoxifen resistance and cell proliferation through the ROS/AKT signalling axis.

## 2 RESULTS

### 2.1 Abnormal expression of ASAH1 in TAMR‐BCs

**FIGURE 1:**
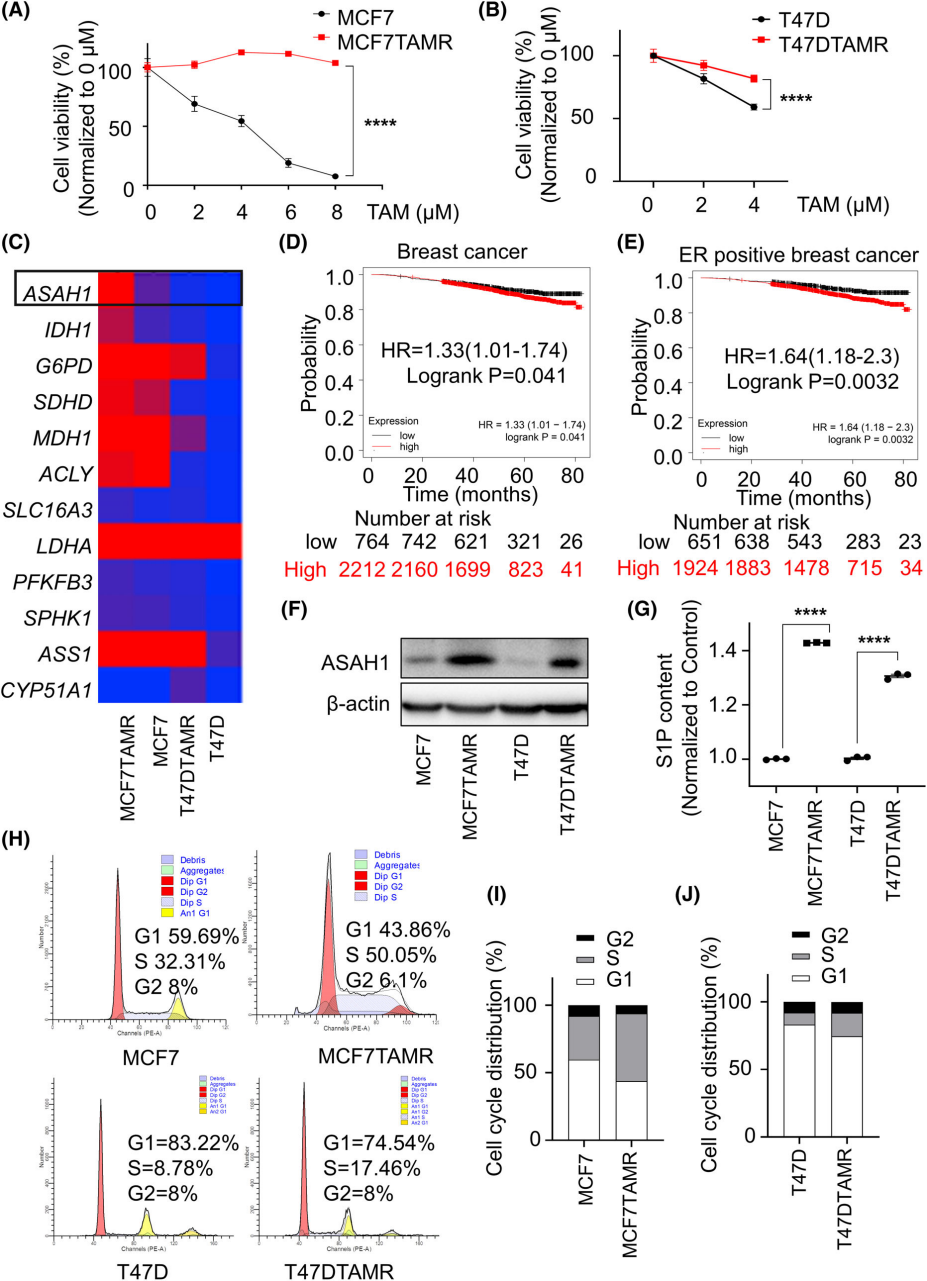
Abnormal expression of ASAH1 in TAMR‐BCs. (A and B) Viability of MCF7 and MCF7TAMR (A), T47D and T47DTAMR (B) cells were detected by MTT assay with tamoxifen at gradient concentrations. (C) Differences in metabolism‐related genes in tamoxifen‐sensitive and drug‐resistant cells according to the GEO database. (D and E) Association between high expression of *ASAH1* and overall survival in clinical samples was analysed through the Kaplan–Meier Plotter website. (F) Immunoblotting was used to verify the difference in ASAH1 expression between cells, and β‐actin was used as an internal reference. (G) Intracellular S1P content in BCs and TAMR‐BCs was detected by ELISA. (H–J) Distribution of the cell cycle in MCF7 (T47D) and MCF7TAMR (T47DAMR) cells was measured (H) and statistically analysed (I and J). BC, breast cancer; ELISA, enzyme‐linked immunosorbent assay; MTT, methyl thiazolyl tetrazolium; TAMR, tamoxifen‐resistant.

Drug resistance of tumour cells has become one of the barriers to clinical therapy, and tamoxifen resistance is a common symptom of ER‐positive BC.^18^ First and foremost, TAMR‐BCs (MCF7TAMR and T47DTAMR) were established by gradient culture of tamoxifen, and showed significantly lower sensitivity to tamoxifen than their control cells (Figure 1A,B). To explore the molecular mechanism of tamoxifen resistance, the changes in metabolism‐related genes were compared in TAMR‐BCs cells and BCs. *ASAH1*, one of the ceramidases in sphingolipid metabolism‐related genes, was abnormally increased in drug‐resistant cells (Figure 1C and Supplementary 1A). Then, we detected overexpression of *ASAH1* in ER+ BC patients relative to normal breast tissues, but this trend was not seen in the other subtypes (Supplementary 1B). To further identify the targeting value of ASAH1 in clinical treatment, the association between *ASAH1* expression and overall survival of patients with BC was analysed through the Kaplan–Meier Plotter website. Overall survival of patients with high *ASAH1* expression (*n* = 2212) was significantly shorter than that of patients with low *ASAH1* expression (*n* = 764, *p* = 0.041) (Figure 1D). Importantly, higher *ASAH1* expression was also accompanied by a lower overall survival rate in patients with ER‐positive BC (*p* = 0.0032) (Figure 1E), indicating that the high expression of *ASAH1* resulted in a poor prognosis for patients, especially for ER‐positive BC. Subsequently, endocrine treated and untreated samples were screened and compared. In the treatment group, the statistical differences in *ASAH1* expression among all patients and ER‐positive patients were *p* = 0.0097 and *p* = 0.0061, respectively, as well as *p* = 0.22 and *p* = 0.049 in the corresponding group without endocrine therapy (Supplementary 1C). Obviously, the high expression of ASAH1 decreased overall survival of BC patients, and was closely related to ER expression and endocrine therapy. Next, we examined the expression of ASAH1 at the cellular level, and ASAH1 was abnormally expressed in TAMR‐BCs including MCF7TAMR and T47DTAMR cells (Figure 1F). Ceramidases are a group of enzymes that catalyse the hydrolysis of ceramide to produce sphingosine, which is subsequently phosphorylated to generate sphingosine 1‐phosphate (S1P). S1P is a biologically active lipid molecule and has been proven to promote cell proliferation.^19^ As expected, the content of S1P was significantly upregulated in both MCF7TAMR and T47DTAMR cells (Figure 1G), and the proportion of S‐phase in MCF7TAMR and T47DTAMR cells was also much higher than that in MCF7 cells (Figure 1H–J). These results indicated a more active sphingolipid metabolism and proliferative phenotype of TAMR‐BC cells.

In brief, ASAH1 was abnormally elevated to promote cell proliferation in TAMR‐BCs. The high expression of ASAH1 represented poor prognosis in clinical samples, and ASAH1 might play an important role in drug resistance of ER‐positive BC cells.

### 2.2 ASAH1‐mediated cell proliferation in TAMR‐BCs

**FIGURE 2:**
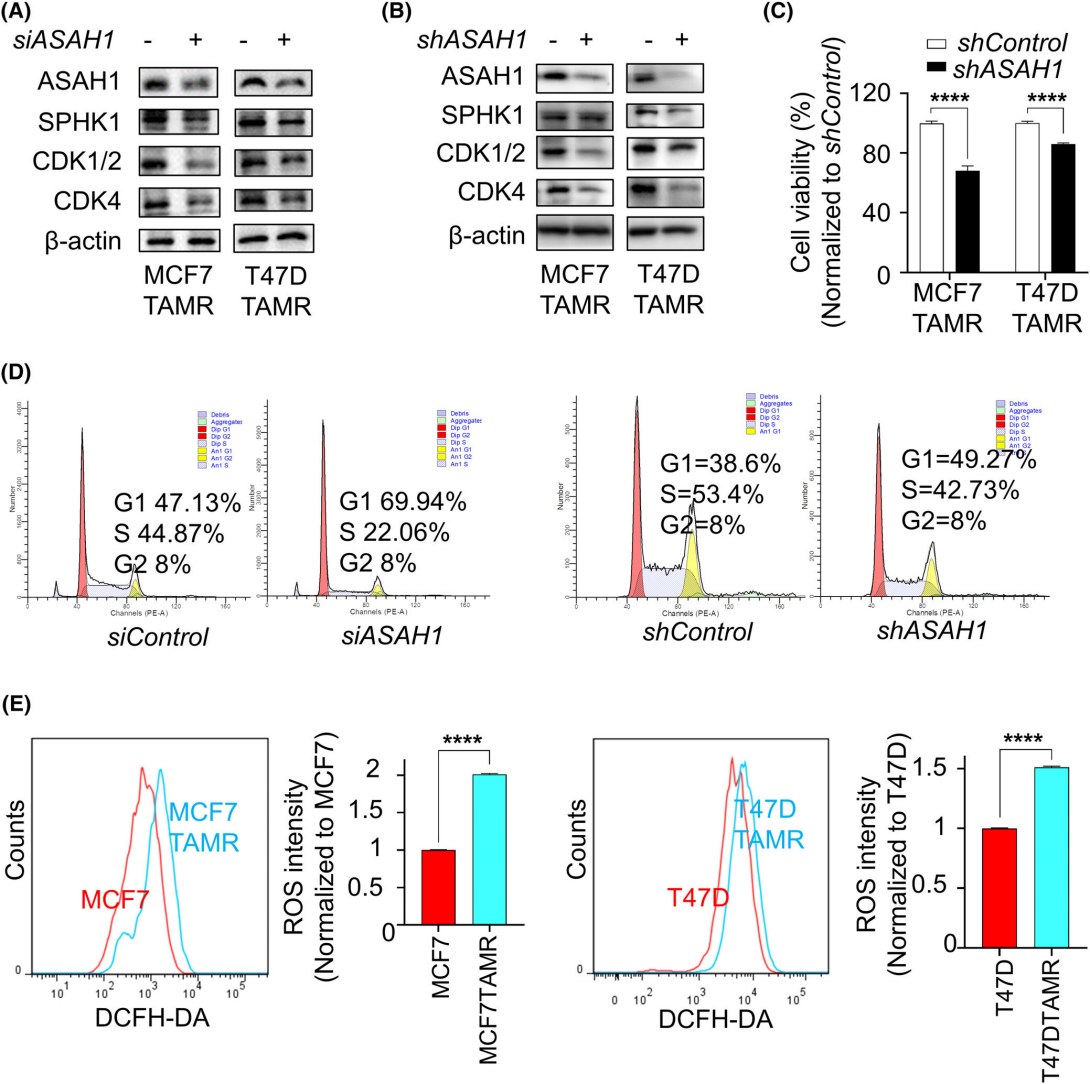
ASAH1‐mediated cell proliferation in TAMR‐BCs. (A and B) Indicated proteins were analysed by immunoblotting in TAMR‐BC cells with or without *ASAH1* knockdown. (C) MTT assay was used to detect the viability of TAMR‐BC cells with or without *ASAH1* knockdown. (D) Cycle distribution of MCF7TAMR cells with or without *ASAH1* knockdown was measured. (E) Intracellular ROS levels were tested in MCF7 (T47D) and MCF7TAMR (T47DAMR) cells. BC, breast cancer; ELISA, enzyme‐linked immunosorbent assay; MTT, methyl thiazolyl tetrazolium; ROS, reactive oxygen species; TAMR, tamoxifen‐resistant.

To determine whether the high expression of ASAH1 is associated with the aberrant proliferative activity of TAMR‐BC cells, we knocked down ASAH1 in MCF7TAMR and T47DTAMR, respectively. The results showed that sphingolipid metabolism levels and cycle‐associated protein expression were reduced regardless of siRNA interference or lentiviral transfection with shRNA (Figure 2A,B). Similarly, knockdown of ASAH1 diminished the survival viability of TAMR‐BC cells (Figure 2C). As stated earlier, S1P plays important roles as second messengers regulating biological processes, such as cell growth, differentiation, migration and apoptosis.^20^ Silenced ASAH1 reduced the content of intracellular S1P (Supplementary 2A), thereby inducing G1 phase arrest in MCF7TAMR and T47DTAMR cells (Figure 2D and Supplementary 2B). However, apoptosis was not observed in cells with ASAH1 silencing (Supplementary 2C). Meanwhile, MCF7 cells displayed higher expression of CDK1/2 and CDK4 after being stimulated with exogenous S1P, suggesting that the activation of sphingolipid metabolism enhanced the proliferation of TAMR‐BCs (Supplementary 2D).

**FIGURE 3:**
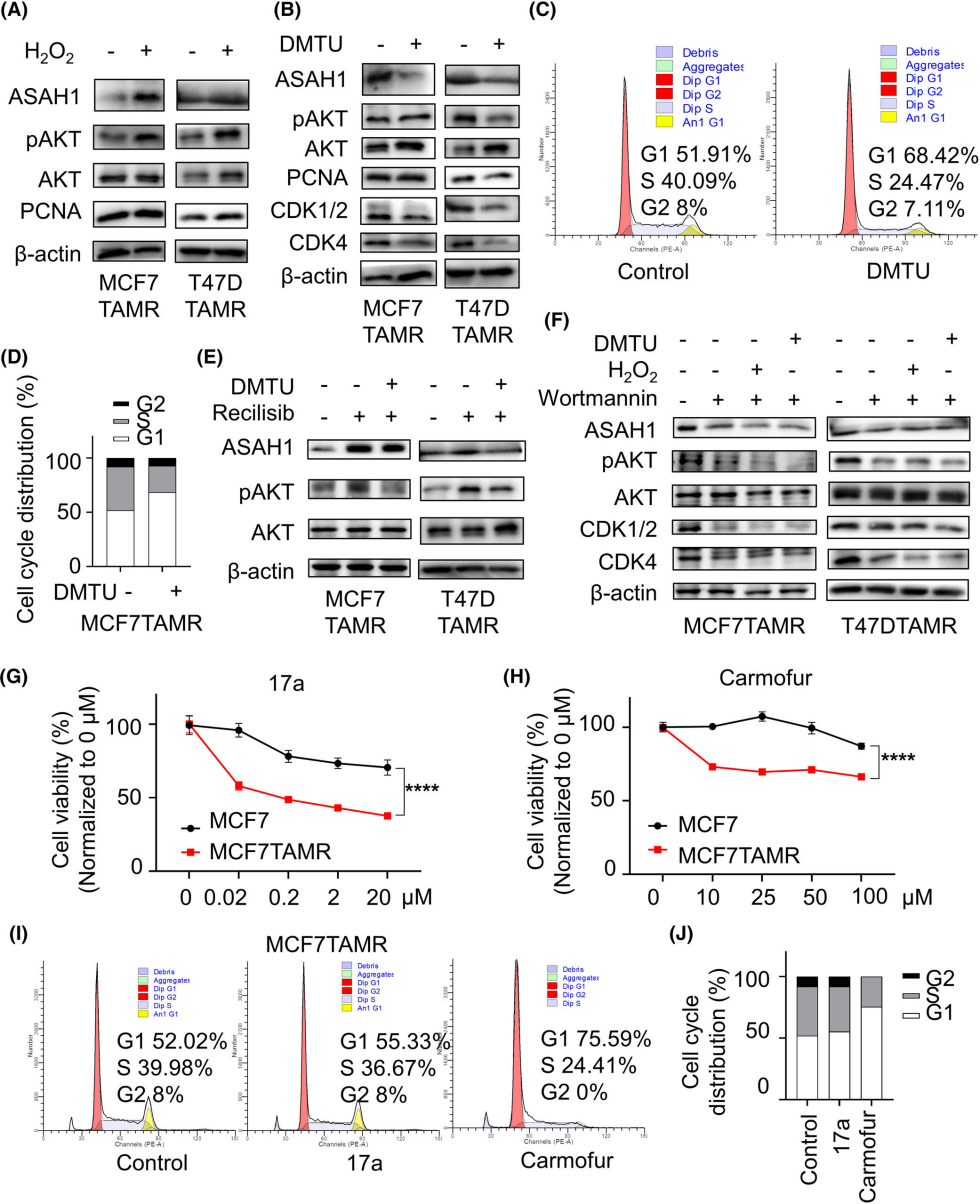
ASAH1 upregulated by ROS/AKT signalling pathway in TAMR‐BCs. (A) Immunoblotting was used to detect the protein changes in TAMR‐BC cells and cells treated with H_2_O_2_. (B) Immunoblotting was used to detect the protein changes in TAMR‐BC cells and cells treated with DMTU. (C and D) Cell cycle distribution of MCF7TAMR cells with or without DMTU treatment was measured (C) and statistically analysed (D). (E) TAMR‐BC cells were treated with the AKT activator recilisib, and then DMTU was added to detect changes in protein expression. (F) TAMR‐BC cells were treated with the AKT inhibitor wortmannin, and then, DMTU or H_2_O_2_ were added to detect the differential expression of proteins. (G and H) Viability of MCF7 and MCF7TAMR cells to 17a (G) or carmofur (H) was detected by MTT assay. (I and J) Cycle distribution of MCF7TAMR cells with 17a or Carmofur was measured (I) and statistically analysed (J). BC, breast cancer; MTT, methyl thiazolyl tetrazolium; ROS, reactive oxygen species; TAMR, tamoxifen‐resistant.

Elevated ROS is one of the characteristics of acquired tamoxifen resistance in ER‐positive BC.^21^ Meanwhile, excessive accumulation of intracellular ROS leads to cell death in tumour cells.^22^ To explore whether tamoxifen resistance was related to the level of intracellular ROS, differences in ROS accumulation between MCF7(T47D) and MCF7TAMR(T47DTAMR) cells were examined. As expected, ROS levels in TAMR‐BC cells were significantly higher than those in control cells (Figure 2E). Next, changes in ROS‐related signalling pathways protein were explored in MCF7 and MCF7TAMR cells. Phosphorylation levels of AKT, rather than STAT1 and STAT3, were significantly activated in MCF7TAMR cells (Supplementary 2E). Thus, we speculated that the abnormal level of ASAH1 might be relative to the ROS/pAKT signal pathway in TAMR‐BC cells. To verify this hypothesis, exogenous H_2_O_2_ treatment was used to increase the level of intracellular oxidative stress.^23^ MCF7TAMR and T47DTAMR cells exhibited activation of pAKT and higher expression of ASAH1 and proliferation level after H_2_O_2_ incubation (Figure 3A and Supplementary 3A). Consistently, when ROS was eliminated with DMTU (dimethylthiourea, the ROS scavenger),^24^ we found that MCF7TAMR and T47DTAMR cells proliferation ability and ASAH1 expression were restrained, as well as AKT signalling was inhibited (Figure 3B–D and Supplementary 3B). Then, the inhibitor (wortmannin^25^) and activator (recilisib^26^) of pAKT were further used to confirm the relationship between pAKT and ASAH1. After cells were treated with recilisib, the expression of ASAH1 increased with the activation of pAKT, but the stimulatory effect of recilisib was partially eliminated with DMTU combined treatment (Figure 3E and Supplementary 3C). Conversely, inhibition of pAKT by wortmannin reduced ASAH1 expression, coupled with decreased expression of cycle‐associated CDK proteins. In addition, when the states of pAKT were suppressed by wortmannin, neither the ROS activator H_2_O_2_ nor the eliminator DMTU affected the expression of pAKT and ASAH1 (Figure 3F and Supplementary 3D). Taken together, ASAH1 upregulated by the ROS/AKT signalling pathway was verified in MCF7TAMR and T47DTAMR cells.

In addition to regulating ASAH1 at the genetic level, two small molecule inhibitors of ASAH1 were used: Carmofur could directly bind to ASAH1 and promote its suppression,^27^ while another antagonist, benzoxazoldone carboxyamide, ARN14974 (17a), is a potent systemic active inhibitor of intracellular acid ceramidase.^28^ Then, we compared the cell viability effects of existing ASAH1 inhibitors in TAMR and control cells. The results showed that 17a and carmofur were higher selective against TAMR cells with high ASAH1 expression, but the inhibitory effect on tamoxifen‐sensitive cells was not significant (Figure 3G,H and Supplementary 3E,F). And both inhibitors did not significantly induce G1‐phase blockade to inhibit the proliferation of drug‐resistant cells. (Figure 3I,J and Supplementary 3G,H).

### 2.3 MCL inhibited cell proliferation by ROS/AKT/ASAH1 signalling in TAMR‐BCs

**FIGURE 4:**
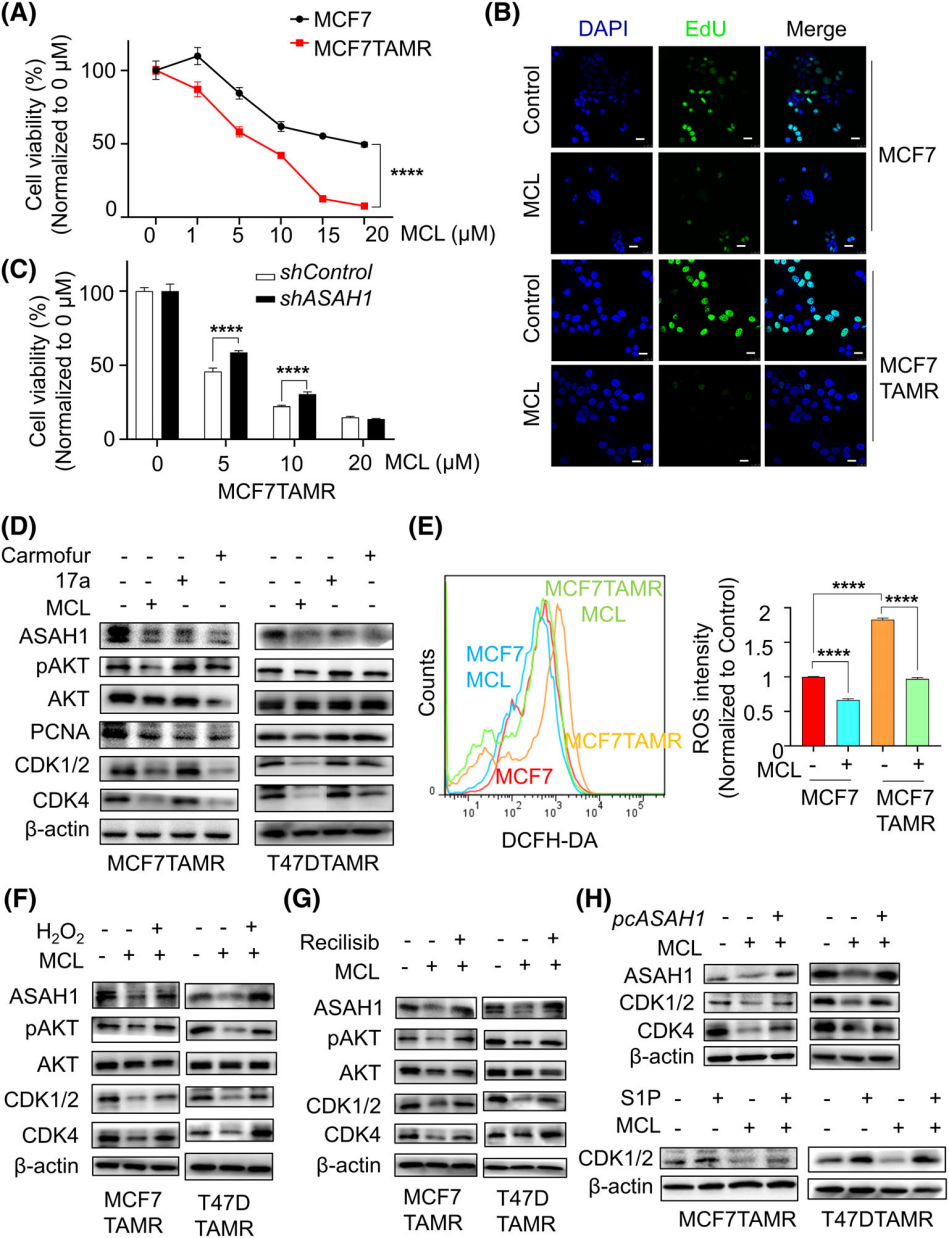
MCL inhibited cell proliferation by ROS/AKT/ASHA1 signalling in TAMR‐BCs. (A) Viability of MCF7 and MCF7TAMR cells to MCL was detected by MTT assay. (B) EdU assay was used to detect proliferating MCF7TAMR cells treated with or without MCL, and DAPI was used for nuclear restaining, bar = 25 μm. (C) MTT assay was used to detect the viability of MCF7TAMR cells with *ASAH1* knockdown or not for MCL. (D) Immunoblotting was used to detect the expression of specified proteins in TAMR‐BC cells treated with MCL, 17a and carmofur. (E) The DCFH‐DA kit was used to detect intracellular ROS levels by flow cytometry in MCF7 or MCF7TAMR cells with or without MCL treatment. (F) TAMR‐BC cells were treated with MCL, and then H_2_O_2_ was added to detect changes in protein expression. (G) TAMR‐BC cells were treated with MCL, and then the AKT activator recilisib was added to detect changes in protein expression. (H) Immunoblotting was used to determine the changes in protein levels in TAMR‐BC cells overexpressing ASAH1 after MCL treatment, MCL treatment alone and control treatment. And, after the cells were treated with MCL, S1P was added exogenously to detect the difference in protein expression among the cells and the control groups. BC, breast cancer; MCL, micheliolide; MTT, methyl thiazolyl tetrazolium; ROS, reactive oxygen species; TAMR, tamoxifen‐resistant.

Given that abovementioned inhibitors did not significantly inhibit the viability of tamoxifen‐sensitive BCs, we aimed to find a small molecule drug that could be selective against both TAMR and tamoxifen‐sensitive cells. Through literature and drug comparisons, we focused on MCL, a drug for leukaemia therapy that could cross the blood–brain barrier and has been shown to inhibit cell proliferation.^29^ Whether MCL is a candidate drug for BC cells was considered. Surprisingly, MCL simultaneously inhibited the viability of BCs and TAMR‐BCs, and the inhibition effect was better than the abovementioned ASAH1 inhibitors, especially for TAMR‐BCs (Figure 4A and Supplementary 4A). Furthermore, treatment with MCL reduced the proportion of cells in the proliferative status (Figure 4B). Meanwhile, MCL led to G1 phase arrest in MCF7TAMR and T47DTAMR cells (Supplementary 4B,C). MCL could inhibit cell viability and proliferation, but does it affect apoptosis? As flow cytometry result, MCL did not induce apoptosis, and the effect was similar to that of *ASAH1* silencing (Supplementary 4D). On the other hand, MCL reduced the S1P content in MCF7TAMR cells (Supplementary 4E). This result led us to link MCL and ASAH1. To explore whether ASAH1 is involved in the antitumor effects of MCL, the gradient concentration of MCL applied to TAMR‐BC cells with or without *ASAH1* knockdown. Interestingly, sensitivity to MCL was significantly decreased in MCF7TAMR and T47DTAMR cells after *ASAH1* knockdown (Figure 4C and Supplementary 4F,G), which indicated that the sensitivity of TAMR‐BCs to MCL was assisted by the high expression of ASAH1. Next, we treated TAMR cells with MCL and ASAH1 inhibitors, respectively, the western blot results showed that MCL inhibited the expression of ASAH1 and PCNA, which was similar to the effect of carmofur and 17a (Figure 4D and Supplementary 5A). A previous study proved that MCL caused oxidative stress and cell death in leukaemia and glioblastoma.^30^ As expected, ROS in both drug‐resistant and non‐drug‐resistant cells were inhibited by MCL (Figure 4E). Likewise, intracellular ROS in MCF7 cells accumulated after treatment with tamoxifen, and excessive ROS were further eliminated with MCL therapy (Supplementary 5B). However, 17a (the inhibitor of ASAH1) treatment did not cause significant changes in ROS levels in MCF7TAMR cells (Supplementary 5C). Besides this, exogenously added H_2_O_2_ or activation of pAKT with recilisib treatment were capable of rescuing MCL‐induced G1 phase arrest, suggesting that the status of ROS/pAKT was crucial for ASAH1 expression and cell proliferation (Figure 4F,G and Supplementary 5D–G). In addition, either ASAH1 overexpression or stimulation with activator S1P was sufficient to reverse the expression of cyclin protein inhibited by MCL treatment in TAMR‐BC cells (Figure 4H), which precisely verified our hypothesis that ASAH1 might be a potential major regulatory target of MCL. In general, MCL suppressed the proliferation of TAMR‐BCs by ROS/AKT/ASAH1 signalling.

### 2.4 MCL restrained ROS by directly binding NRF2/KEAP1

The results above demonstrated that MCL restrained ASAH1 expression through the ROS/pAKT pathway. It has been reported that MCL inhibited the biological function of thioredoxin reductase by covalently binding to it, which contributed to radiosensitization and induced ROS‐mediated apoptosis in HeLa cells.^31^ However, MCL reduced oxidative stress in BCs and TAMR‐BCs in our study. How does MCL interact with redox stress? Genes related to ROS production or clearance pathways were monitored in cells.^32^ There was no significant difference in the expression of *NOX1‐4* genes, only expression of *NOX2* was consistent with our study. That is, the *NOX2* gene enhanced in MCF7TAMR cells relative to MCF7, and was inhibited by MCL in both types of cells (Supplementary 6A). On the other hand, the genes of the clearance pathway, named *SOD*, *CAT* and nucleafactorerythroid‐2‐related factor 2 (*NFE2L2*), did not change in different groups, but the expression difference of heme oxygenase‐1 (*HMOX1*) was significant, which was increased in MCF7TAMR cells and abnormally enhanced by MCL treatment in MCF7 and MCF7TAMR cells (Supplementary 6A). As one of the key antioxidant proteins, the expression of HO‐1 may be an important reason for restraining cellular oxidative stress.

**FIGURE 5:**
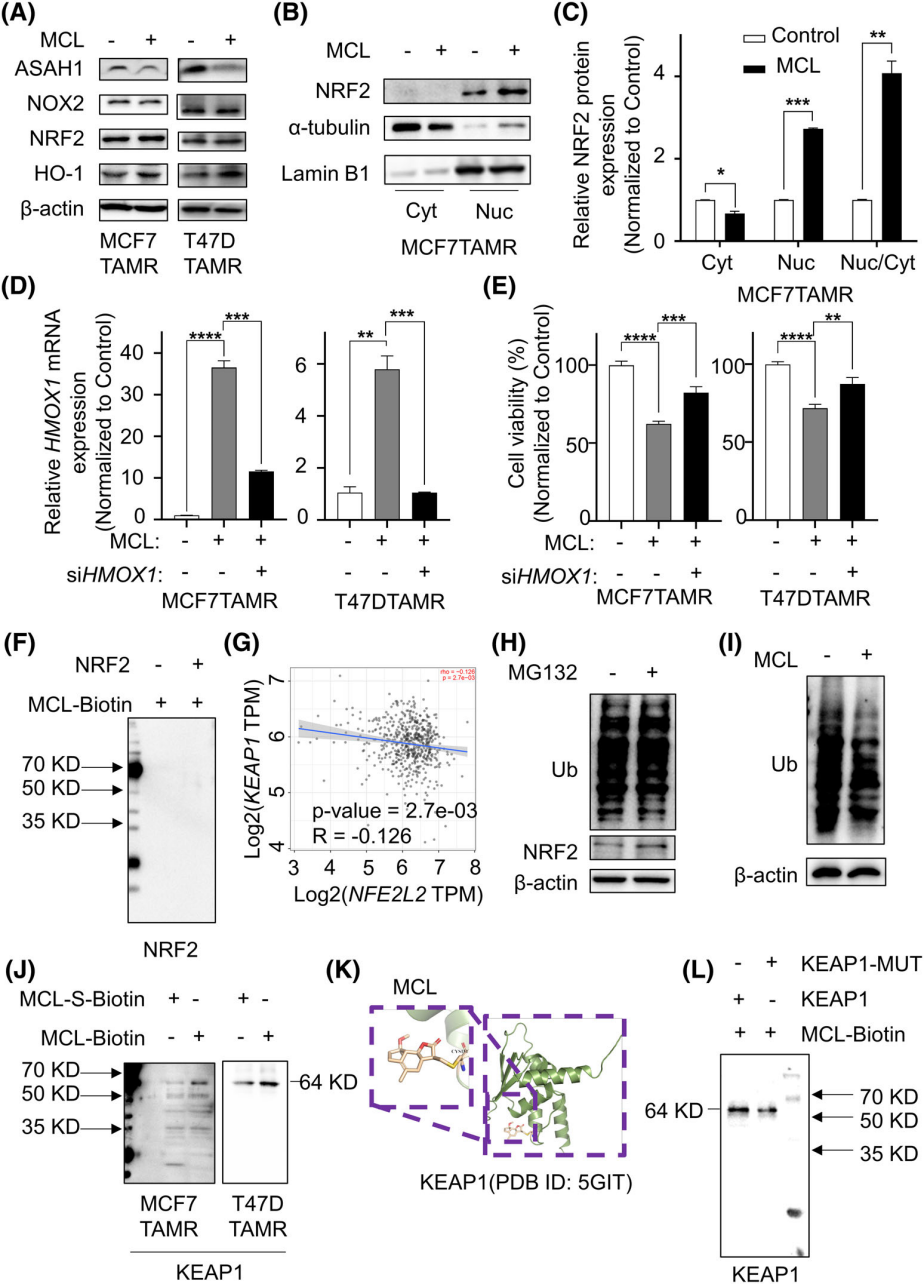
MCL restrained ROS by directly binding KEAP1. (A) Immunoblotting was used to detect the expression of ROS‐related proteins in TAMR‐BC cells treated with or without MCL. (B and C) Protein expression and nuclear translocation of MCF7TAMR cells were detected by cytoplasmic separation before and after MCL treatment. (D) qPCR was used to detect the expression of *HMOX1* genes in TAMR cells treated with MCL or MCL plus *siHMOX1*. (E) MTT assay was performed to monitor the viability of TAMR cells with MCL or MCL plus *siHMOX1*. (F) MCL‐Biotin was used to incubate purified NRF2 protein and the negative control group (PBS), and NRF2 was detected by immunoblotting. (G) The correlation between *NFE2L2* and *KEAP1* gene expression in breast cancer was analysed by TIMER. (H and I) MCF7TAMR cells were treated with the proteasome inhibitor MG132 (H) or MCL (I) to detect the ubiquitin level and the expression of the specified protein. (J) TAMR‐BC cell lysates were pulled down by MCL‐Biotin or MCL‐S‐Biotin, and KEAP1 was detected by immunoblotting. (K) CovDock was used for covalent docking between the small molecule drug MCL and the KEAP1 protein. (L) Purified KEAP1 and its mutant, whose Cys‐151 was mutated to A‐151, and the expression of the protein pulled down by MCL‐Biotin was detected by immunoblotting. BC, breast cancer; MCL, micheliolide; MTT, methyl thiazolyl tetrazolium; PBS, phosphate‐buffered saline; ROS, reactive oxygen species; TAMR, tamoxifen‐resistant.

HO‐1 is an important antioxidant enzyme that mainly catalyses the catabolism of heme into ferrous iron, carbon monoxide and biliverdin, and the transcriptional activity of HO‐1 is directly regulated by NRF2.^33^ MCL slightly activated the expression level of NRF2, but the expression of HO‐1 was significantly increased (Figure 5A). However, as a transcription factor of HO‐1, the difference of NRF2 did not seem to be enough to support such a significant change of HO‐1. Nucleoplasmic fractionation experiments were performed to detect whether MCL could achieve the regulation of HO‐1 through NRF2. MCL promoted the expression of NRF2, especially enhanced its nuclear translocation, and the nucleoplasmic ratio of NRF2 protein increased by about 4 times (Figure 5B,C). MCL treatment caused a significant increase in the gene level of *HMOX1*. However, when *HMOX1* knocked down with siRNA in addition to MCL treatment (Figure 5D), that could alleviate the reduction of cell viability caused by MCL (Figure 5E). HO‐1 can function as a downstream effector molecule of NRF2. Therefore, we speculated that NRF2 played an important role in MCL‐mediated ROS elimination.

The 11,13‐double bond of MCL, acts as a reactive Michael receptor and forms a covalent bond with cysteine residues of its target protein, which is essential for its anticancer activity.^34^ To explore the mechanism between MCL, NRF2 and ROS, a biotin tag was added to the C domain of MCL (MCL‐biotin), and the 11,13‐double bond was changed into a single bond to prepare MCL‐S‐biotin (Supplementary 6B). Then, we purified NRF2 protein and co‐incubated it with MCL‐Biotin. Surprisingly, there was no direct interaction between MCL and NRF2 (Figure 5F). Considering that MCL treatment did not influence the transcription level of NRF2 in MCF7TAMR cells (Supplementary 6A), we hypothesized that MCL targeted NRF2 by post‐transcriptional modification. The literature illustrates that most of the functional effects of small molecule compounds on NRF2 achieved through KEAP1.^35^, ^36^, ^37^ KEAP1 traps NRF2 in the cytoplasm and promotes its degradation by the 26S proteasome.^35^ Whether GEPIA (Supplementary 6C) or TIMER (Figure 5G) online analysis showed a significantly negative correlation between *NFE2L2* and *KEAP1* gene expression in invasive BC samples. Therefore, is the expression of NRF2 in BC affected by the level of ubiquitination? As expected, NRF2 was enhanced upon treatment with the proteasome inhibitor MG132 (Figure 5H). However, MCL significantly attenuated the ubiquitination level of proteins (Figure 5I). Importantly, MCL‐biotin was able to immunoprecipitate with KEAP1 (Figure 5J), leading us to wonder whether MCL directly bound with KEAP1 to inhibit the ubiquitination of NRF2 and stabilize its expression. Then, we queried the amino acid composition of KEAP1 on the UniProt website and found 27 cysteines, which were likely to covalently bind to MCL (Supplementary 6D). Covalent modification of KEAP1 on Cys‐151 results in a conformational change in KEAP1 to dissociate the KEAP1‐ubiquitin ligase complex, and the simulating score of covalent binding of MCL and KEAP‐Cys151 was found to be consistent with Britannin^38^ (Figure 5K and Supplementary 6E). Next, we purified KEAP1 and its mutant protein, whose cysteine‐151 was mutated to alanine, and the co‐immunoprecipitation (co‐IP) results showed that the binding of the mutant protein to MCL was significantly weakened (Figure 5L). Therefore, MCL stabilized the antioxidant regulatory protein NRF2 by directly binding to KEAP1 and promoting its nuclear translocation, thereby attenuating oxidative stress in MCF7TAMR cells, and ultimately suppressing tumour proliferation through the ROS/pAKT/ASAH1 pathway.

### 2.5 ACT001 inhibited the growth activity of TAMR‐BCs in vivo

**FIGURE 6:**
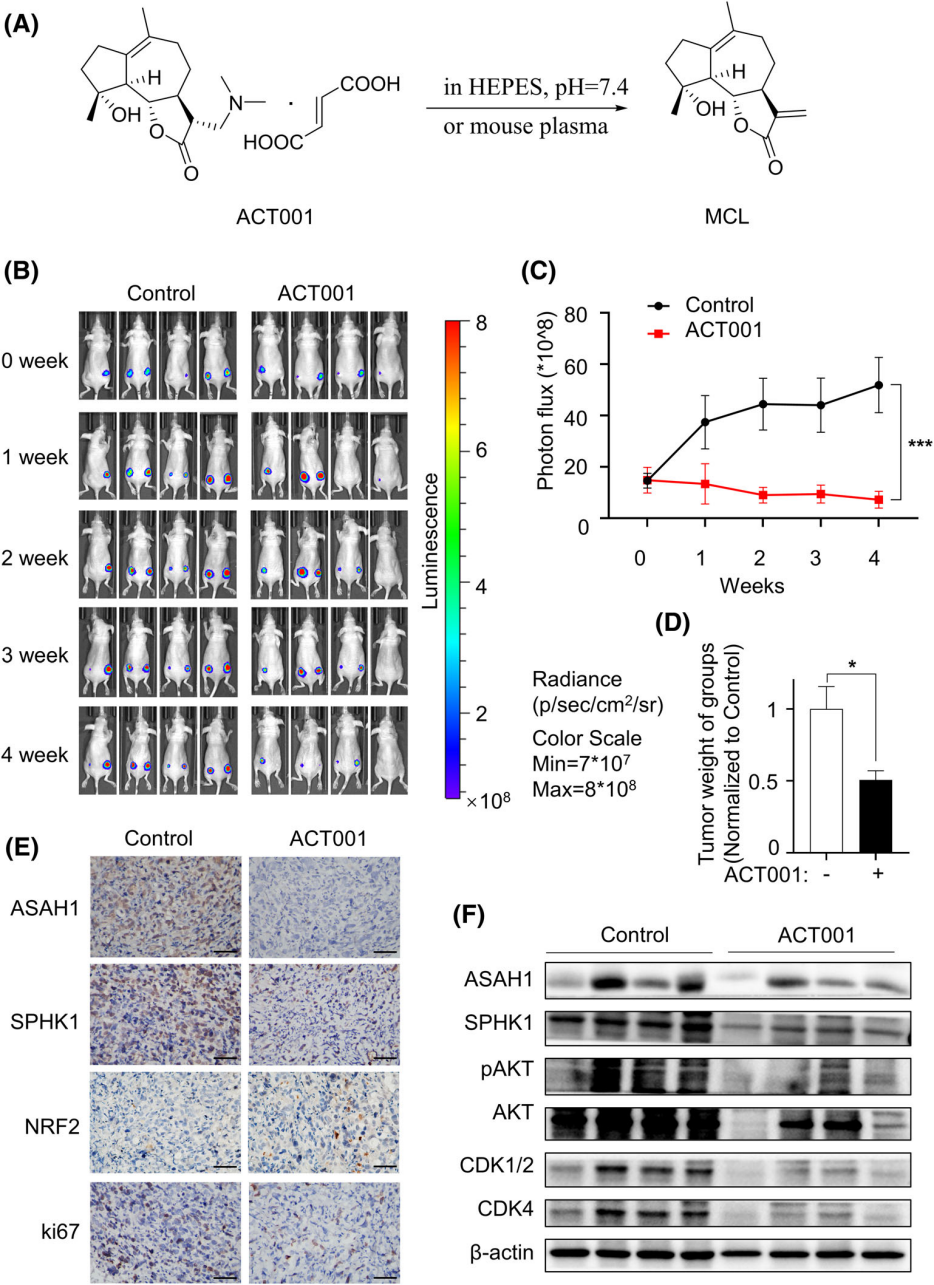
ACT001 inhibited the growth activity of TAMR‐BCs in vivo. (A) Diagram of ACT001 releasing MCL in mouse plasma or (N‐2‐hydroxyethylpiperazine‐N‐2‐ethane sulfonic acid) HEPES. (B and C) Nude mice were randomly divided into the ACT001 group and the control group after 10 days of tumour bearing with MCF7TAMR‐Luc cells. Fluorescence images (B) were taken at 0, 1, 2, 3 and 4 weeks, and statistical analysis was performed (C). (D) The weight of tumour tissues in each group was analysed statistically. (E) IHC assay was performed to detect the indicated proteins in the ACT001 administration and control groups, bar = 50 μm. (F) Immunoblotting was used to determine the expression differences of proteins in each group. BC, breast cancer; IHC, immunohistochemistry; MCL, micheliolide; TAMR, tamoxifen‐resistant.

ACT001, a prodrug of MCL, could release MCL slowly and continuously in plasma (Figure 6A) and was chosen to perform animal experiments. MCF7TAMR‐Luc cells (1 × 10^7^) were subcutaneously injected into the backs of nude mice to establish tumour models. Compared with the control group, ACT001 treatment displayed a lower bioluminescence intensity growth rate and diminished tumour weight, but had no obvious effect on the body weight of mice (Figure 6B–D and Supplementary 6F), indicating that ACT001 significantly inhibited MCF7TAMR tumour growth in vivo. At the end of the experiment, tumours were removed for further analysis. Immunohistochemistry (IHC) staining of the administration group showed that the positive rate of nuclear proliferation antigen (ki67) was significantly reduced, as well as the expression of ASAH1/SPHK1 related to sphingolipid metabolism, while the expression of NRF2 was increased (Figure 6E and Supplementary 6G). The immunoblotting results further confirmed that the levels of ASAH1/SPHK1, AKT phosphorylation and cycle‐related proteins were also suppressed in the ACT001 group (Figure 6F and Supplementary 6H). Overall, these in vivo results corroborated the potential role of MCL in suppressing tumour proliferation, which was consistent with in vitro studies.

## 3 DISCUSSION

ER‐positive BC accounts for approximately 70% of the total number of BC patients, and tamoxifen is the main drug for endocrine therapy in this type of patient, but about half of patients exhibit clinical tamoxifen resistance.^18^ In the context of the tumour microenvironment, both tumour cells and drug‐resistant cells are accompanied by tumour metabolic reprogramming to accelerate cancer proliferation and survival, and the importance of lipid metabolism in the TAMR cancer model has already been proposed.^39^ However, studies on the relationship between drug resistance and sphingolipid metabolism represented by ceramides are not clear.

Ceramides and S1P are key lipid molecules of sphingolipid metabolism. On the one hand, ceramide mediates cell stress damage and apoptosis, while S1P mediates cell growth and survival; thus, the balance between the two lipids is essential for cell fate determination.^19^ Recent studies have shown that ASAH1 is associated with the development of drug resistance in tumour cells. In head and neck cancer, inhibition of ASAH1 promoted cisplatin‐induced apoptosis in vitro and in vivo.^40^ In addition, studies in GBMs^41^ and prostate cancer^42^ further suggested that upregulation of ASAH1 conferred resistance to radiation through an altered sphingolipid metabolism pathway. However, there is no current study on the association between ASAH1 and tamoxifen resistance in BC. We found that ASAH1 was abnormally activated in TAMR cells, which were more sensitive to ASAH1 inhibitor treatment. It has been reported that the proximal promoters c‐Jun and c‐Fos, members of activating protein 1 (AP‐1), are recruited to the irradiated nucleus and participate in the transcriptional expression of ASAH1.^42^ ASAH1 further promotes the nuclear export of PTEN via the downstream product S1P, thereby promoting cell proliferation and tumour therapeutic resistance.^43^ According to our results, elevated ASAH1 in TAMR‐BCs was regulated by activated ROS/pAKT signalling. AKT is a major effector of oxidative stress,^44^ and PI3K/AKT activation has also been reported to be involved in the pro‐survival of TAMR cells,^45^ which is consistent with our results. Tamoxifen‐sensitive cells stimulated by an AKT activator exhibited increased AKT phosphorylation accompanied by ASAH1 activation, and the expression of pAKT and ASAH1 was further elevated due to the high oxidative stress in TAMR‐BCs. Therefore, there may be positive feedback in the AKT/ASAH1 regulatory network that accelerates tumour progression.

Small molecule inhibitors targeting ASAH1 have been continuously developed, but most are still under in vitro experimental studies. The clinical drug carmofur has no significant therapeutic effect in liver cancer and has been withdrawn due to its toxicity.^11^ Thus, it is urgent to perform already discovered drugs in clinical trials or to find other alternative drugs targeting ASAH1. In this study, the responsiveness of MCF7TAMR and T47DTAMR cells was basically consistent, but MCF7TAMR cells were more sensitive to ASAH1 inhibitor relative to their parental cells than T47DTAMR; we hypothesized that this was due to tumour heterogeneity. Surprisingly, both MCF7TAMR and T47DTAMR were more sensitive to MCL, which suggests that MCL has a better targeting therapeutic effect on TAMR cells.

MCL, as a marketed drug, can cross the blood–brain barrier and reach the lesion by oral administration. In our study, MCL inhibited the activation of pAKT/ASAH1 by reducing ROS accumulation in ER‐positive BC cells, especially TAMR cells, thereby suppressing tumour proliferation. Contrary to our conclusions, MCL^31^ and ACT001^46^ have been reported to induce oxidative stress in tumour cells, which may be due to the heterogeneity of tumour cells, resulting in inconsistent responses to the drug among different tumour types. A study revealed that MCL‐induced oxidative stress was mediated by GSH, and the combination of MCL with a GSH biosynthesis inhibitor (L‐buthionine sulfoximine) is a potent therapeutic intervention for leukaemia and glioblastoma.^30^ Therefore, we wondered whether the combination of MCL and tamoxifen has greater potential for TAMR‐BCs. Unfortunately, neither MCL alone nor the combination of MCL and tamoxifen‐induced apoptosis in TAMR‐BCs, but the combination significantly restrained cell viability and induced G1 phase arrest (Supplementary 7). Therefore, from the perspective of inhibiting tumour proliferation, MCL and tamoxifen have the possibility of clinical combined application.

MCL did not induce ROS‐dependent apoptosis. In contrast, MCL significantly alleviated oxidative stress in ER‐positive cells and TAMR cells. We compared the changes of key molecules about the ROS generation and clearance pathway in TAMR cells with or without MCL treatment. The results demonstrated that MCL stabilized the expression of the antioxidant protein NRF2 and promoted its nuclear translocation by directly binding to KEAP1. KEAP1 is a cysteine thiol‐rich sensor of redox damage and a part of the E3 ubiquitin ligase.^47^ Under normoxic conditions, KEAP1 binds to NRF2 and is inactivated by ubiquitination and proteasome degradation. However, under oxidative stress conditions, NRF2 disassociates from KEAP1 and then translocates to the nucleus, where it activates the transcription of several antioxidant enzymes.^48^, ^49^ Specifically HO‐1, NRF2/HO‐1 plays an important role in tumour proliferation and drug resistance.^50^ In our study, we found that MCL could restrain the elevated ROS level of TAMR cells, and promote the nuclear translocation of NRF2 to increase the transcriptional activation of *HMOX1*. *HMOX1* knocked down reversed the cell viability of MCL inhibition. In fact, while exploring the regulation of cellular oxidative stress, the binding of MCL/HO‐1 was also detected. MCL‐Biotin could not be immunoprecipitated with HO‐1 (data not shown). Consistent with our results, Fisetin inhibits migration of BC cells via inducing HO‐1 and elevating NRF2 expression in nuclear fraction.^51^ However, the specific molecular mechanisms involved in the activation of NRF2/HO‐1 signalling by MCL, whether it focuses only on HO‐1 regulation or there are other cofactors involved, need to be further investigated. In addition, the NRF2 activator DMF (dimethylfumarate) has been approved by the FDA as a new oral agent for the treatment of patients with relapsing multiple sclerosis.^52^ In our study, we demonstrated that MCL was able to directly bind to KEAP1 but had no direct binding possibility with NRF2 through pull‐down experiments, thus maintaining the stability of NRF2 in cells treated with MCL. How does MCL achieve the combination with KEAP1? The literature shows that Cys‐151 is one of the three major cysteine sensors of KEAP1 and maintains an active state.^35^ Covalent modification on Cys‐151 results in a conformational change of KEAP1, which leads to the dissociation of the KEAP1‐ubiquitin ligase complex.^36^ Meanwhile, α‐methylene‐γ‐lactone, as an effective group of MCL, could target the cysteine sulfhydryl of proteins.^53^ Therefore, we speculated that MCL could bind to the cysteine sensor of KEAP1 to dissociate NRF2/KEAP1 and to stabilize NRF2 expression. This assumption was further confirmed by the reduced binding force of MCL and KEAP1 with the Cys‐151 mutation. However, whether the binding between MCL and Cys‐151 of KEAP1 is singular or synergistic with other cysteines remains to be further studied.

Cellular oxidative stress is a complex regulatory network in cancers, and it is characterized by an imbalance between the production of ROS and antioxidant defence mechanisms.^54^ Antioxidants play an auxiliary role in the prevention and treatment of tumours.^55^ However, a broad range of antioxidants promote melanoma migration and metastasis.^56^ In this study, we innovatively found that MCL could stabilize the expression of NRF2 by binding to KEAP1, thereby activating the cellular antioxidant system in tamoxifen‐resistant cells. This applies together in TAMR‐BCs and in BCs with higher intracellular oxidative stress than normal cells, but the role of MCL in other therapeutic modalities requires further study.

## 4 CONCLUSION

**FIGURE 7:**
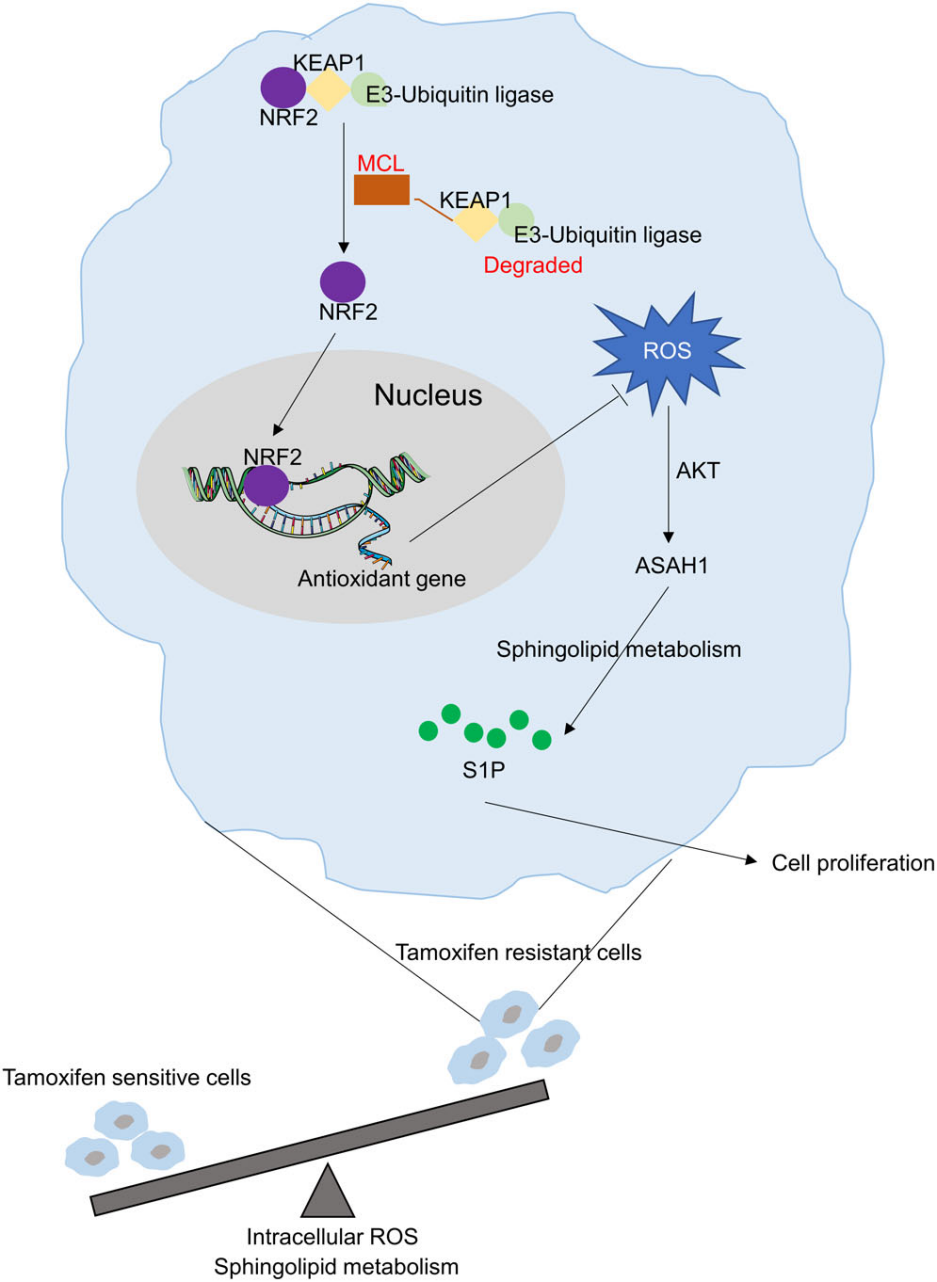
Schematic diagram of molecular mechanism.

In general, TAMR‐BCs showed abnormal accumulation of ROS, which further activated the pAKT/ASAH1 signalling axis‐mediated cell proliferation. MCL stabilized and activated the antioxidative function of NRF2 by directly targeting KEAP1 and dissociating it from NRF2, thereby attenuating oxidative stress and suppressing the ROS/AKT/ASAH1‐mediated cell proliferation (Figure 7). Thus, targeting the ROS/AKT/ASAH1 signalling pathway represents a potential therapy for inhibiting TAMR‐BC.

## 5 MATERIALS AND METHODS

### 5.1 Cell lines and culture

MCF7 and T47D ER‐positive BC cells were successively treated with 0.1, 0.5 and 1 μM tamoxifen (MedChemExpress, USA), and the cells were cultured for 2 months at each concentration. TAMR cell lines MCF7TAMR and T47DTAMR were successfully established after stimulation with tamoxifen for 6 months. MCF7 and T47D cells were cultured in 1640 medium with 1% penicillin‐streptomycin (Solarbio, China) and 10% fetal bovine serum (FBS; HyClone, USA), MCF7TAMR and T47DTAMR cells were additionally added 1 μM tamoxifen to maintain the drug‐resistant properties. HEK293T and HEK293F cells were maintained in Dulbecco's modified Eagle's medium. All cells were incubated in an incubator at 37°C and 5% CO_2_.

### 5.2 Reagents

In addition to the indicated drugs of gradient concentrations, the main drugs and concentrations used in this paper were MCL (Shangdeyaoyuan, China, 5 μM), S1P (MedChemExpress, USA, 5 μM), H_2_O_2_ (Sigma, USA, 30 μM), DMTU (Sigma, USA, 20 mM), recilisib (MedChemExpress, USA, 5 μM), wortmannin (MedChemExpress, USA, 5 μM), MG132 (Cayman, USA, 5 μM), tamoxifen (TAM, MedChemExpress, USA, 2 μM), ARN14974 (17a, Cayman, USA, 2 μM) and carmofur (Cayman, USA, 5 μM). All reagents were initially prepared with dimethyl sulfoxide (DMSO) and diluted with medium.

### 5.3 Small RNA interference and plasmid construction

Small RNA interference (siRNA, 5 μM) technology was achieved by liposome transfection for 48 h, and the targeting sequence of si*ASAH1* (GTTCCATGGTACACCATAA) and si*HMOX1* (CGATGGGTCCTTACACTCA) were designed and obtained from RIBOBIO (China).

Short hairpin RNA (shRNA, 4 μg) was transfected with pLP1, pLP2 and VSV‐G; thus, lentivirus was packaged in 293T cells. Lentivirus was transfected into MCF7TAMR cells, following selected with puromycin, MCF7TAMR‐shASAH1 cells were acquired. The shRNA sequence was designed as GTTCCATGGTACACCATAA and synthesized by Tsingke Biotechnology (China).

### 5.4 Cell proliferation assay

The methyl thiazolyl tetrazolium (MTT) assay was used to detect cell viability. A total of 5000 cells/well were plated in a 96‐well plate, and the cells were treated with specified concentrations of drugs in medium containing 1% FBS. Then, the supernatant was discarded after incubation with MTT (Solarbio, China) solution at 37°C for 2 h, and 100 μL of DMSO was added to the wells. The absorbance was detected at 492 nm.

### 5.5 Real‐time qPCR and primers

The total mRNA of cells was isolated with a kit (TIANGEN, China), and cDNA was acquired via reverse transcription of 1 μg of mRNA with a First‐Strand cDNA synthesis kit (Thermo, USA). PCR was performed with cDNA (0.5 μg), SYBR mix (TIANGEN, China), H_2_O and primers. The primers were designed as follows:

*ASAH1*,

ATTGGCCCCAGCCTACTTTAT (F), CCCTGCTTAGCATCGAGTTCAT (R). *NOX1*,

GCCAATGTTGACCCAAGGATTTT (F), GGTTGGGGCTGAACATTTTTC (R). *NOX2*,

AACGAATTGTACGTGGGCAGA (F), GAGGGTTTCCAGCAAACTGAG (R). *NOX3*,

CGTGGCGCATTTCTTCAACC (F), GCTCTCGTTAGGGGTGTTGC (R).

*NOX4*,

TGTGCCGAACACTCTTGGC (F), ACATGCACGCCTGAGAAAATA (R).

*SOD*,

GGTGGGCCAAAGGATGAAGAG (F), CCACAAGCCAAACGACTTCC (R). *CAT*,

TGGAGCTGGTAACCCAGTAGG (F), CCTTTGCCTTGGAGTATTTGGTA (R). *NFE2L2*,

TCCAGTCAGAAACCAGTGGAT (F), GAATGTCTGCGCCAAAAGCTG (R). *HMOX1*,

AAGACTGCGTTCCTGCTCAAC (F), AAAGCCCTACAGCAACTGTCG (R).

*β‐actin*,

CACCATTGGCAATGAGCGGTTC (F), AGGTCTTTGCGGATGTCCACGT (R).

### 5.6 Immunoblotting analysis

The total proteins of tumour tissues and cells were lysed and extracted. The proteins were separated with a 12% separating gel. After membrane transformation, blocking and antibody incubation, fluorescence imaging was performed by the HRP method. Antibodies against ASAH1, SPHK1, SPHK2, S1PR1, S1PR3, PCNA, Bim, cl‐PARP, cl‐caspase3, AKT, pAKT, α‐tubulin, Lamin B1 and pSTAT1 were purchased from Cell Signaling Technologies (USA). Antibodies against CDK1/2, CDK4, CDK2, STAT1, STAT3, pSTAT3, NOX2, NRF2, HO‐1, Keap1, Ub and β‐actin were acquired from Santa Cruz (USA). Phosphorylated proteins were normalized to total proteins, nuclear and plasma proteins normalized to Lamin B1 and α‐tubulin, respectively, and other proteins normalized to β‐actin. All protein content was expressed in relative units in comparison with control samples loaded on each gel. ImageJ software was used in protein quantification.

### 5.7 Flow cytometry analysis

ROS (DCFH‐DA), JC‐1 (mitochondrial membrane potential), apoptosis and cell cycle were performed according to the instructions of the kit (MultiSciences, China), and the results of ROS, JC‐1 and apoptosis were analysed using Flow Jo V10 software, while the cell cycle was analysed by ModFit software.

### 5.8 Kits

NADP^+^/NADPH was detected with a kit (Beyotime, China), and the absorbance was measured at 450 nm with a microplate reader.

Cytosolic and nuclear extractions were performed with a kit (Beyotime, China), the efficiency of separation was measured by immunoblotting analysis, the cytoplasm was indicated by α‐tubulin, and the nucleus was indicated by Lamin B1.

An EdU assay (Beyotime, China) was used to monitor the status of cell proliferation, and a TUNEL (TdT‐mediated dUTP nick end labeling) assay was performed on sections of tumour tissue according to the instructions (Yeasen, China). The cell or tissue section was counterstained with haematoxylin, and the microscopic images were obtained by confocal microscopy at a magnification of 630×.

### 5.9 Enzyme‐linked immunosorbent assay

The cells were lysed after three cycles of freezing and thawing, and then the content of S1P in the cells was monitored by ELISA (Becton, Dickinson and Company, USA).

### 5.10 Co‐IP assay

MCL‐Biotin and MCL‐S‐Biotin were purchased from a company (Shangdeyaoyuan, China). The lysed cells were evenly divided into two groups, and each group (1 μg) was incubated with MCL‐Biotin (100 μM) or MCL‐S‐Biotin (100 μM) at 4°C overnight. Biotin‐linked magnetic beads were resuspended with products incubated overnight and rotated at 4°C for 4 h. After washing four times, the magnetic beads were resuspended in 1× loading buffer and boiled at 95°C for 5 min, and the supernatant sample was taken for immunoblotting analysis.

### 5.11 Site‐directed mutagenesis, protein expression and purification

Full‐length NRF2, KEAP1 and KEAP1‐MUT coding genes were cloned into the pTT5 vector (NovoPro, China) for expression of the N‐terminal His‐tagged recombinant proteins. KEAP1 mutant, KEAP1‐MUT generated by QuikChange site‐directed mutagenesis kit (Stratagene, USA) according to wild‐type KEAP1. The above three plasmids were transfected into HEK293F cells for expression for 5 days at 37°C. The cell supernatant was harvested, and His‐tagged proteins were purified using HisTrap HP columns (GE Healthcare, USA).

### 5.12 IHC staining

The obtained tumour tissues were dipped in wax, embedded and sectioned for IHC to monitor the changes in protein expression in tissues. After hydration, the tissue sections were incubated with 1% Triton for 15 min to break the membrane, and then 3% H_2_O_2_ was used to remove endogenous peroxidase. The tissues were blocked with goat serum for 1 h after antigen repaired. After incubated with primary and secondary antibodies and DAB (3,3‐N‐diaminobenzidine tetrahydrochloride, Solarbio, China) solution, the slices were counterstained with DAPI, sealed with neutral resin and imaged with light microscopy at 400×. Four different locations were randomly selected, the percentage area of positive staining was quantified in the IHC staining using ImageJ software.

### 5.13 Animal experiments

The ethics committee for animal use at the medical college of Nankai University approved the animal experiments. BALB/c nude mice were used as the experimental animal model. MCF7TAMR cells (2*10^6^) were subcutaneously implanted into both flanks of mice, which were randomly divided into two groups (five mice per group) after 1 week. The administration group was gavaged with ACT001 (400 mg/kg) once every 2 days, while normal saline was administered to the control group. Primary tumours were assessed with bioluminescence imaging every week. Tumour tissues were harvested for the following experiments.

### 5.14 Statistical analysis

All data are shown as the mean ± SEM of at least three independent experiments and were analysed using GraphPad Prism 8.0 statistical software unless otherwise stated. Student's *t* test and two‐way ANOVA were used to compare the significance between groups, and *p* < 0.05 was considered significant.

## AUTHOR CONTRIBUTIONS

X. H., Y. Z. and Y. L. designed and performed all the experiments in vivo and in vitro. Z. L., Y. Z., D. S. and Y. L. were responsible for data analysis. S. Z, Z. F. and C. L. contributed to the supervision and writing guidance of the study. X. H., S. Z. and Z. L. were involved in writing the manuscript. C. W., S. Z., C. L. and D. S. provided project reviewing and funding support. All authors participated in the experimental process, approved the final version of the manuscript and had full access to the data.

## CONFLICT OF INTEREST STATEMENT

The authors declare no conflicts of interest.

## Supporting information


**Data S1.** Supporting Information.

## Data Availability

All data generated or analysed during this study are included in this published article and are available from the corresponding author upon reasonable request.
