# Supplementary material for: MCL restrained ROS/AKT/ASAH1 pathway to therapy tamoxifen resistance breast cancer by stabilizing NRF2
Source: Cell Prolif. 2024 Jun 26;57(11):e13700. doi: 10.1111/cpr.13700 (PMC11533064; doi:10.1111/cpr.13700)
Supplement: Supplementary file 1 — Data S1. Supporting Information. [file CPR-57-e13700-s001.pdf]

supplementary materials

Supplementary 1

A

Gene	MCF7	MCF7TAMR	Ratio	Gene	T47D	T47DTAMR	Ratio
ASAH1	28.3296	101.702	3.6	*ASAH1*	0.0001	5.69	56900.0
IDH1	21.1877	57.7791	2.7	*PFKFB3*	0.14	12.33	88.1
G6PD	198.558	495.918	2.5	*CYP51A1-AS1*	1.61	24.91	15.5
SLC16A3	7.74306	17.8233	2.3	*ASS1*	21.56	289.12	13.4
SDHD	58.627	75.3342	1.3	*MDH1*	3.59	39.16	10.9
PFKFB3	15.3187	20.8037	1.4	*IDH1*	1.04	10.4	10.0
LDHA	349.019	437.22	1.3	*G6PD*	7.61	71.99	9.5
MDH1	107.868	137.77	1.3	*ACLY*	1.77	9.42	5.3
SPHK1	21.4911	23.4291	1.1	*SLC16A3*	2.06	10.72	5.2
ACLY	86.7543	73.2779	0.8	*SDHD*	1.52	7.75	5.1
ASS1	239.672	141.415	0.6	*SPHK1*	2.91	13.24	4.5
CYP51A1-AS1	0.154593	0.034929	0.2	*LDHA*	87.57	369.06	4.2

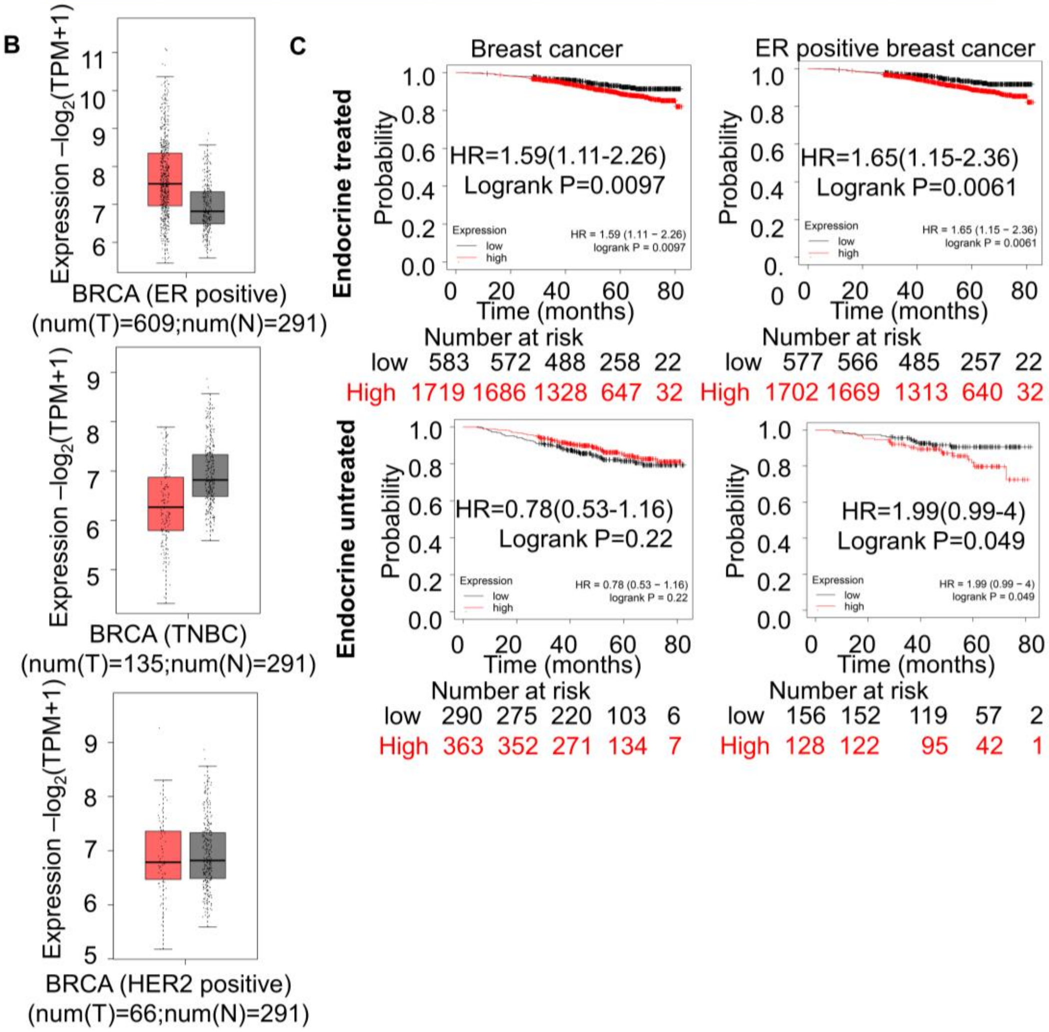

Supplementary 1

A, Differences in metabolism-related genes in tamoxifen-sensitive and drug-resistant cells according to the GEO database. B, The TCGA database was used to analyze the gene expression of *ASAH1* in normal individuals (n=291) and ER positive patients (n=609), in normal individuals (n=291) and TNBC patients (n=135) or in normal individuals (n=291) and HER2 positive patients (n=66). C, Association between high expression of ASAH1 and overall survival in clinical samples was analyzed through the Kaplan–Meier Plotter website.

Supplementary 2

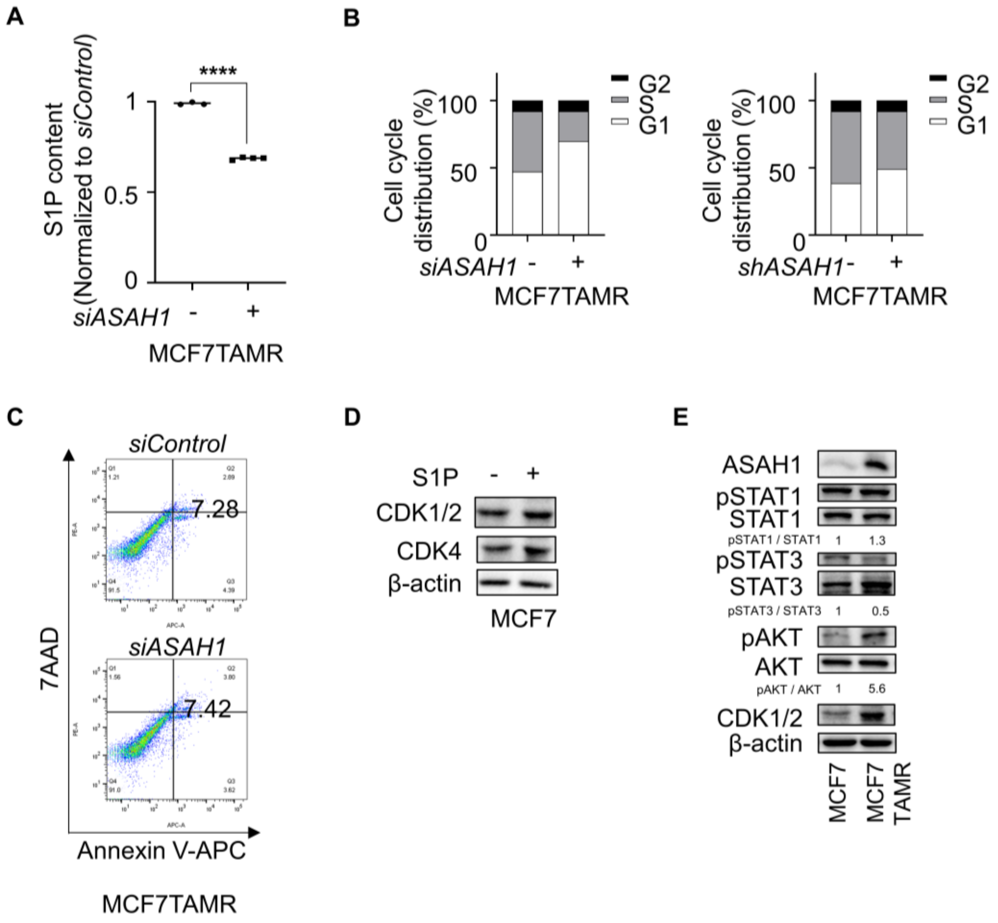

Supplementary 2

A, Intracellular S1P content was detected in MCF7TAMR cells with or without *ASAH1* knockdown. B, Cycle distribution was statistically analyzed in MCF7TAMR cells with or without *ASAH1* knockdown. C, Apoptosis of MCF7TAMR cells with or without *ASAH1* knockdown was detected by flow cytometry. D, Changes in proteins were detected in cells treated with exogenous S1P for 8 h and in the control group. E, Immunoblotting was used to detect the protein changes in MCF7 and MCF7TAMR cells.

Supplementary 3

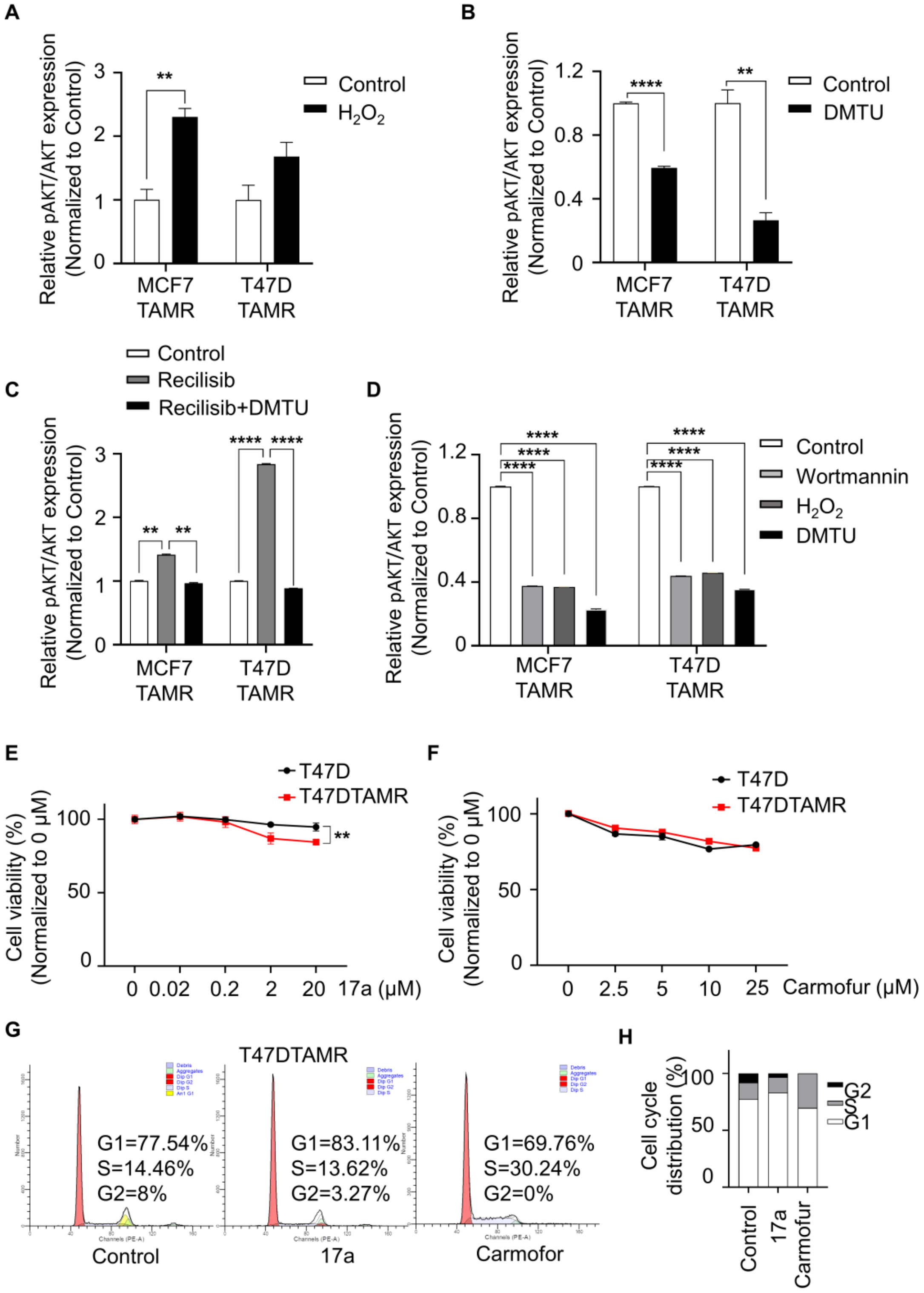

Supplementary 3

A-D, Densitometry was performed to analyze protein expression by ImageJ software. E and F, Viability of T47D and T47DTAMR cells to 17a (E) or carmofur (F) was detected by MTT assay. G and H, Cycle distribution of T47DTTAMR cells with 17a or Carmofur was measured (G) and statistically analyzed (H).

Supplementary 4

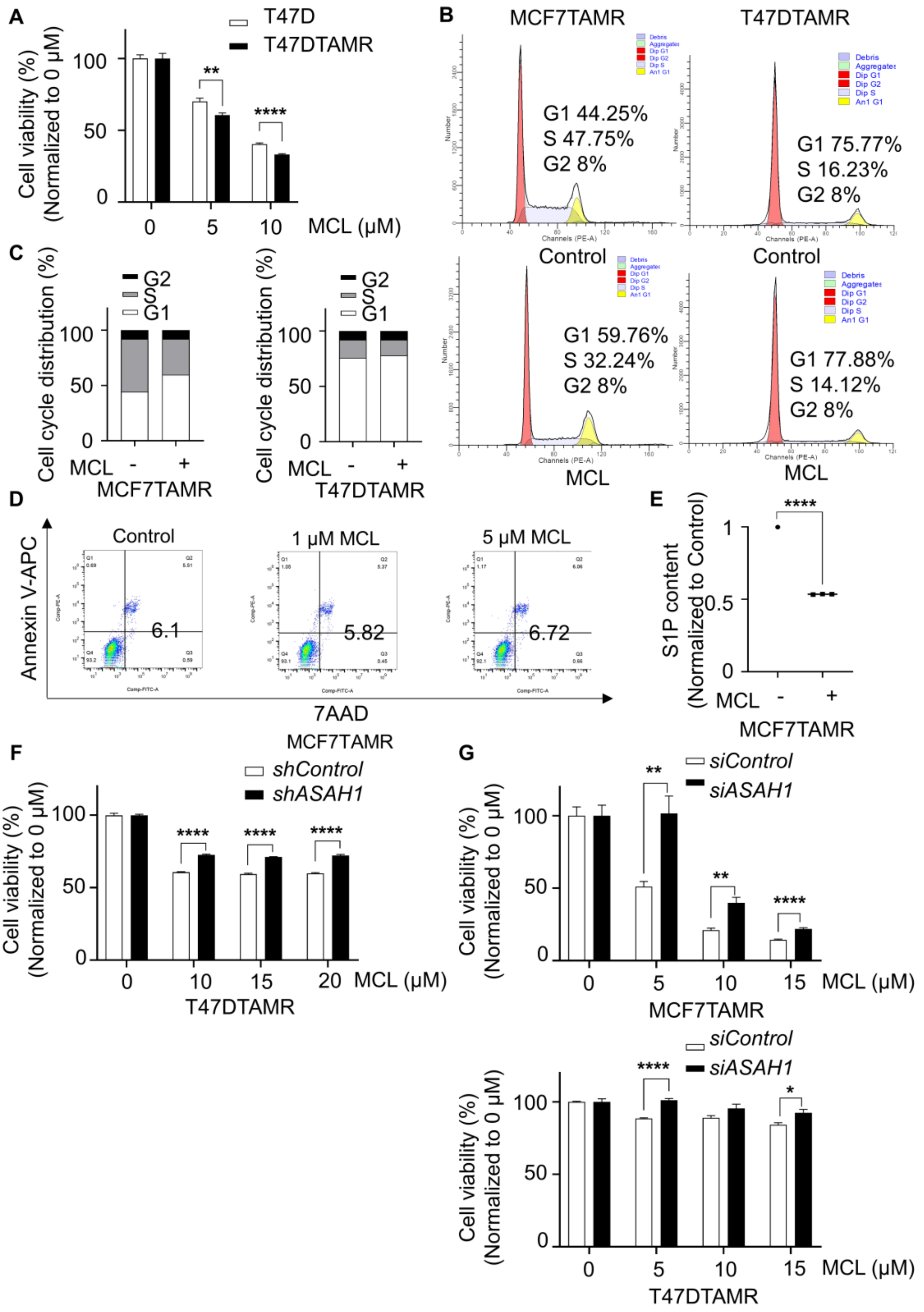

Supplementary 4

A, Viability of T47D and T47DTAMR cells to MCL was detected by MTT assay. B and C, Cell cycle distribution of TAMR-BC cells with or without MCL treatment was measured (B)

and statistically analyzed (C). D, Apoptosis of MCF7TAMR cells treated with or without MCL was detected by flow cytometry. E, Intracellular S1P content was detected in MCF7TAMR cells with or without MCL treatment. F, MTT assay was used to detect the viability of T47DTAMR cells with *shASAH1* or not for MCL. G, MTT assay was used to detect the viability of TAMR-BC cells with *siASAH1* or not for MCL.

Supplementary 5

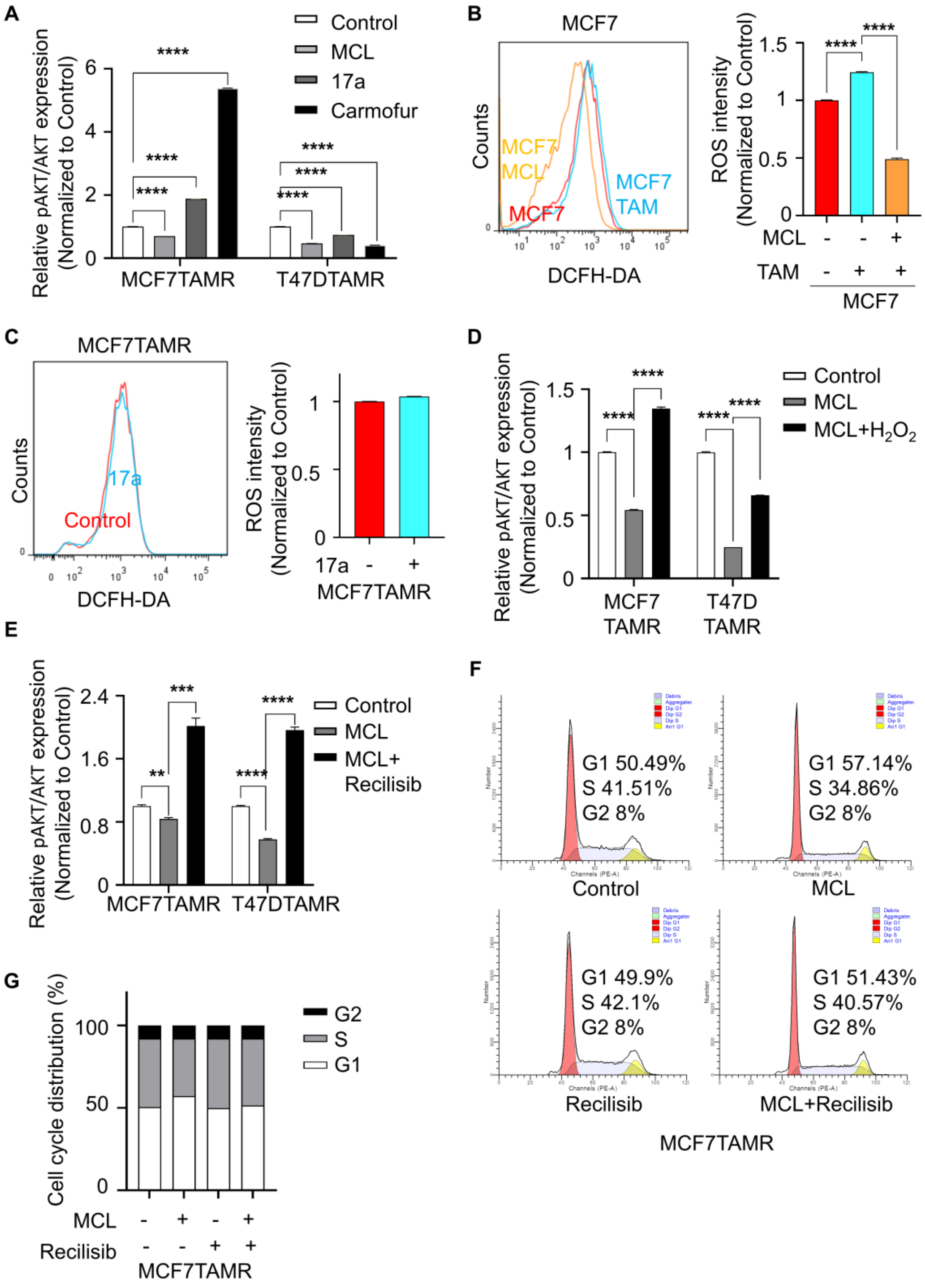

Supplementary 5

A, Densitometry was performed to analyze protein expression by ImageJ software. B, Intracellular ROS levels were tested in MCF7 cells, cells treated with tamoxifen or cells treated with tamoxifen combined with MCL. C, Intracellular ROS levels were tested in MCF7TAMR cells with or without 17a treatment. D and E, Densitometry was performed to analyze protein expression by ImageJ software. F and G, Cell cycle distribution was tested in MCF7TAMR cells treated with MCL accompanied by recilisib treatment (F) and statistically analyzed (G).

Supplementary 6

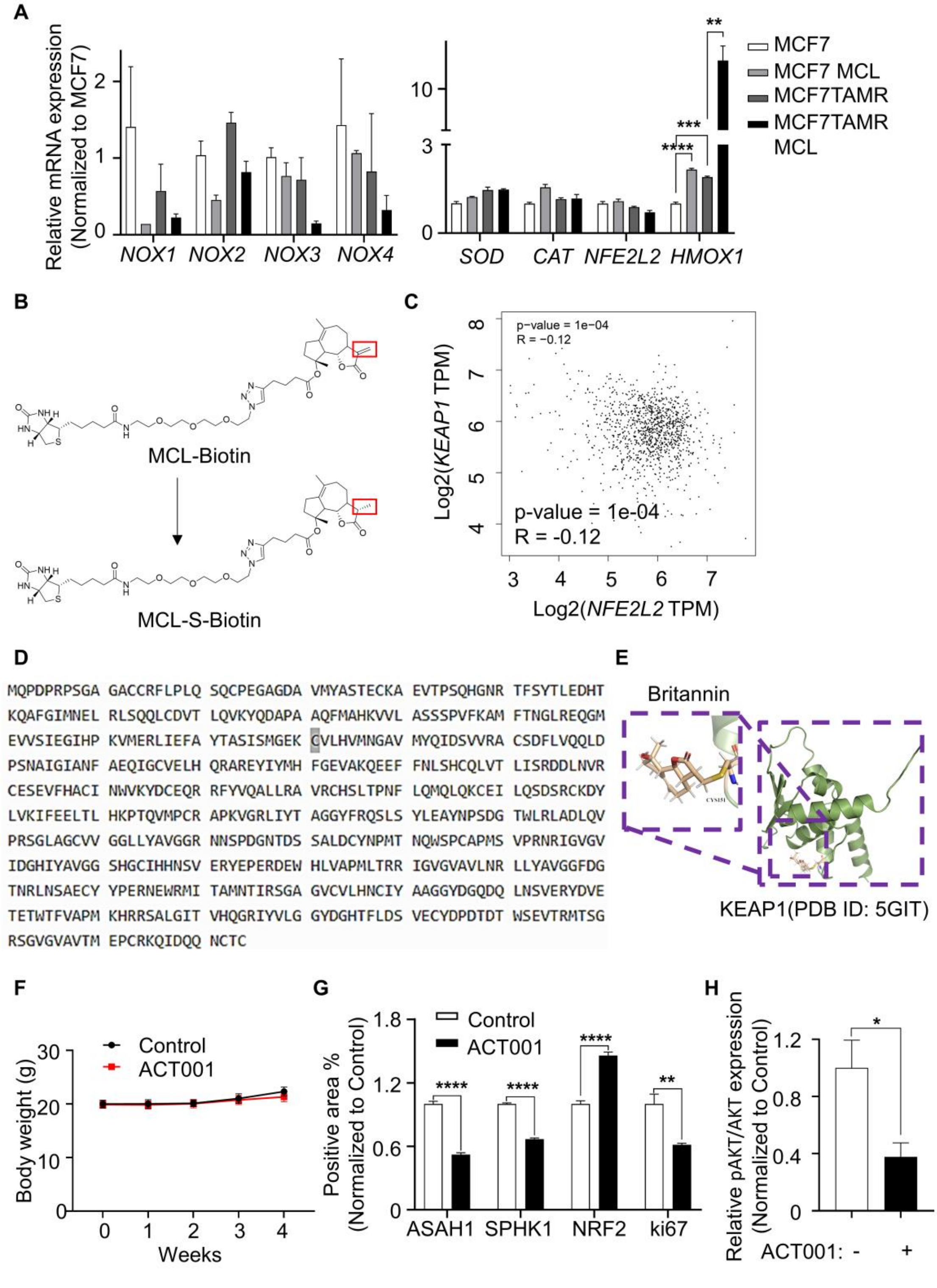

Supplementary 6

A, qPCR was used to detect the expression of ROS-related genes in MCF7 and MCF7TAMR cells treated with or without MCL. B, Schematic diagram of the molecular structure of MCL binding with biotin (MCL-Biotin) and morphism of the active double bond to single bond (MCL-S-Biotin). C, The correlation between *NFE2L2* and *KEAP1* gene expression in breast cancer was analyzed by GEPIA. D, Amino acid composition of the KEAP1 protein. E, CovDock was used for covalent docking between the small molecule drug Britannin and the KEAP1 protein. F, Changes in body weight of mice during drug administration. G, The positive area in tissue sections was quantified with normalization to control by ImageJ software. H, Densitometry was performed to analyze protein expression by ImageJ software.

Supplementary 7

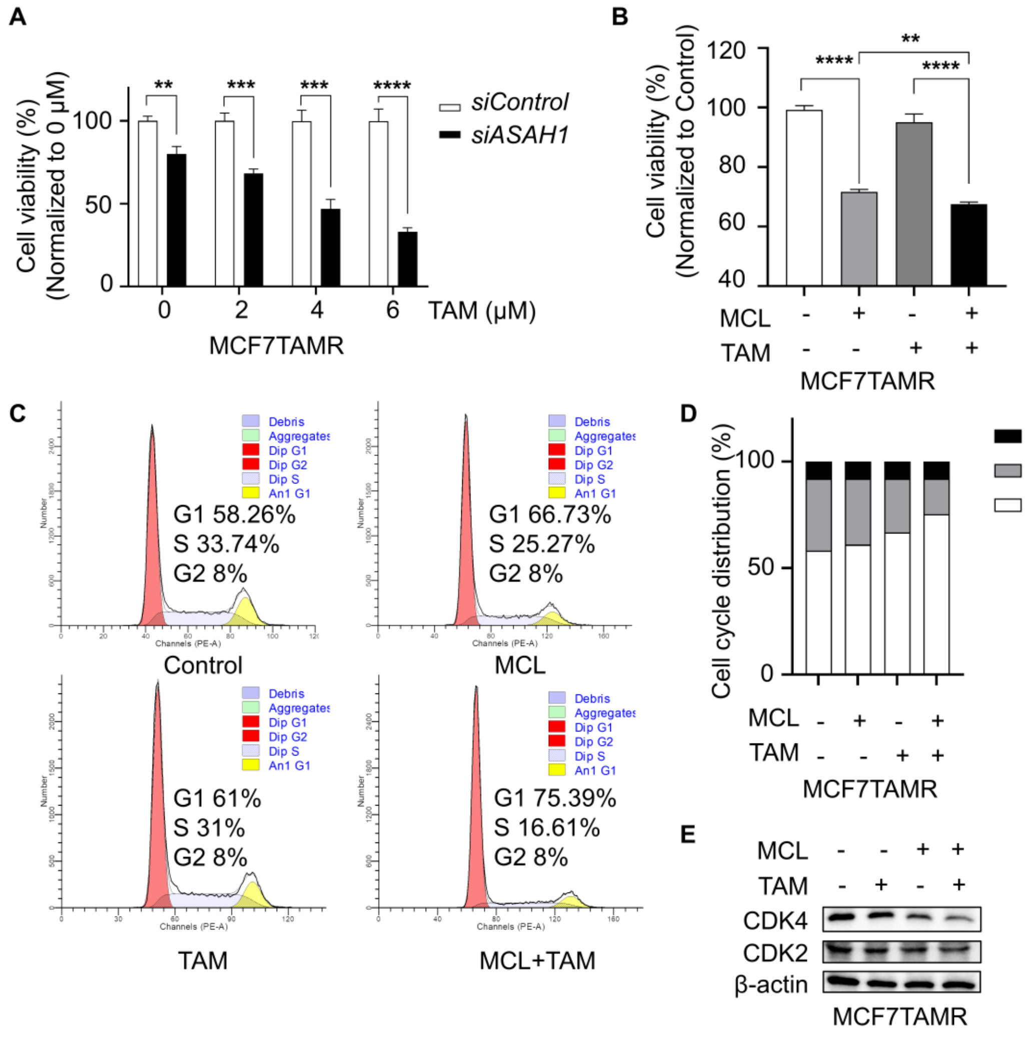

Supplementary 7

A, MTT assay was used to detect the viability of MCF7TAMR cells with or without *ASAH1* knockdown after treatment with tamoxifen at a gradient concentration. B, The sensitivity of MCF7TAMR cells to MCL was detected by MTT assay in the presence or absence of tamoxifen. C and D, Cell cycle distribution of MCF7TAMR cells with or without MCL treatment was measured (C) and statistically analyzed (D) in the presence or absence of tamoxifen. E, Immunoblotting was used to detect differences in cell cycle-related proteins in MCF7TAMR cells treated with MCL in the presence or absence of tamoxifen.
